# Physical learning environments’ supportiveness to innovative pedagogies: students’ and teachers’ experiences

**DOI:** 10.1007/s10984-022-09433-x

**Published:** 2022-11-08

**Authors:** S. Baars, G. L. M. Schellings, J. P. Joore, P. J. V. van Wesemael

**Affiliations:** 1 Eindhoven University of Technology, Eindhoven, The Netherlands; 2 NHL Stenden University of Applied Sciences, Leeuwarden, The Netherlands; 3 Delft University of Technology, Delft, The Netherlands

**Keywords:** Higher education, Innovative pedagogy, Physical learning environment, Psychosocial learning environment, Teachers’ and students’ experiences

## Abstract

Research into the relationship between innovative physical learning environments (PLEs) and innovative psychosocial learning environments (PSLEs) indicates that it must be understood as a network of relationships between multiple psychosocial and physical aspects. Actors shape this network by attaching meanings to these aspects and their relationships in a continuous process of gaining and exchanging experiences. This study used a psychosocial-physical, relational approach for exploring teachers’ and students’ experiences with six innovative PLEs in a higher educational institute, with the application of a psychosocial-physical relationship (PPR) framework. This framework, which brings together the multitude of PLE and PSLE aspects, was used to map and analyse teachers’ and students’ experiences that were gathered in focus group interviews. The PPR framework proved useful in analysing the results and comparing them with previous research. Previously-identified relationships were confirmed, clarified, and nuanced. The results underline the importance of the attunement of system aspects to pedagogical and spatial changes, and of a psychosocial-physical relational approach in designing and implementing new learning environments, including the involvement of actors in the discourse within and between the different system levels. Interventions can be less invasive, resistance to processes could be reduced, and innovative PLEs could be used more effectively.

## Introduction

In recent years, many educational institutions have invested in adapting their school buildings to innovative pedagogies, either on their own initiative or stimulated by government programs such as Building for the Future (UK) and Building Environment Revolution (AU). These school building policies are based on the assumption that innovative psychosocial learning environments (PSLE) impose different requirements on the physical learning environment (PLE) as described in the conceptual literature. This literature advocates spatial concepts for school building structures in which clusters of diverse learning spaces, rather than classrooms, are the building blocks of school buildings, arguing that these spatial concepts optimally support the varied learning activities as propagated by innovative pedagogies (Fisher, 2005; Nair et al., 2013). Conversely, traditional school building structures could hinder the implementation of such pedagogies (Cleveland & Fisher, 2014). Because of this assumption, many traditional school buildings have been reconstructed or replaced. However, innovative PLEs do not always deliver on their promises (Veloso & Marques, 2017). There is a great need for empirically-acquired insight into how innovative PLEs are related to innovative pedagogical practices.

Our earlier literature review (Baars et al., 2020) shows that an increasing number of empirical studies confirm a relationship between innovative PLEs and innovative PSLEs, but also that this relationship is more complex than would be expected (Mulcahy, 2016). An innovative PLE does not automatically lead to an innovative PSLE. Alignment of the intended use of the innovative PLE with teachers’ pedagogical vision and practice appears to be decisive for its success (Daniels et al., 2019). This underlines the importance of empirically examining how innovative PLEs are understood, used, and experienced by teachers and students.

## Theoretical background

Designing and implementing an innovative learning environment involve many participants, each contributing at different levels, over a long period of time and with each participant constructing a personal representation of the PSLE, the PLE, and their interrelations. Because of the complexity of the process, these representations can differ, or even contradict each other on certain aspects. Although different names are used in the literature, three representations are generally distinguished: the 'intended', 'implemented' and 'achieved' representation (Van den Akker, 2013). The intended representation refers to staff’s ideals and formal policies. The implemented representation refers to the PSLE-PLE teachers’ perceptions and practices. The attained representation refers to students’ experiences and performance. The literature frequently addresses discrepancies between the intended PSLE-PLE of policy makers at school level, and the implemented PSLE-PLE of teachers at class level. Mulcahy et al. (2015) and Daniels et al. (2019) argue that this can be traced back to the rational approach dominating school building policies in which spatial and psychosocial environments are considered to be two separate worlds. The rational approach suggests a direct causal relationship between both. The PLE’s design could be derived from the innovative pedagogical practice and, conversely, these practices would derive from the innovative PLE. This binary approach overlooks the complexity of the PSLE-PLE relationship. Users must recognise and deploy the potential of the PLE for their pedagogical practice in a process of experience-building and sharing (Fenwick et al., 2012). Therefore, the PLE cannot be separated from psychosocial practice, because both the PSLE and the PLE develop in relation to each other in a continuous process by which users—as individuals and as a group—continuously assign meaning to a multitude of psychosocial and physical elements, as well as to their assumed and experienced relationships (Dovey & Fisher, 2014; Mulcahy, 2016). This psychosocial-physical relational approach emphasises the social-semiotic construction of the PSLE-PLE relationship, rather than suggesting the PLE has a meaning in itself with a predictable impact on the PSLE. In this approach—as applied in this current study—the continuously-developing relations between these elements are points of departure (Dovey & Fisher, 2014; Mulcahy et al., 2015). Therefore, research into the PSLE-PLE relationship demands a comprehensive conceptualisation of all elements of the psychosocial-physical learning environment in an overarching framework, including all PLE an PSLE elements, to enable a systematic analysis and comparison of the empirically-observed representations and relations.

### PPR framework: categorising the involved psychosocial and physical aspects

In our previous study, we developed a PSLE-PLE relationship (PPR) framework that guided the methodology of the present study (Baars et al., 2020). In the framework, we applied the authoritative conceptualisations of Moos (1980)—for the PSLE—and Barrett et al. (2015) —for the PLE—to categorise all aspects as identified by previous research to be involved in the PSLE-PLE relationship (see Table 1).

**Table 1:** PPR Framework

Aspect	Description
*Psychosocial learning environment*	
Personal development	Directions of personal growth and self-enhancement
Open-endedness	The extent to which learning is oriented on predefined, uniform learning products or on an open-ended, personalised learning processes
Relevance and integration	The extent to which the learning content is segregated into subject matters, or integrated in a multidisciplinary context, with a recognisable relevance and connection with the students’ world
Environmental interaction	The extent to which the school’s environment is involved in the learning process and learning outcomes
Relationships	Nature and intensity of personal relationships within the learning environment
Teacher support	The extent to which the teacher is interested in—and responds to—students’ learning needs
Student cooperation	The extent to which students collaborate in constructing knowledge by discussing and assessing the viability of ideas with peers
Group cohesiveness	The extent to which students mutually respect each other, are friendly and supportive, know each other/feel known, connected and accepted
Student involvement	The extent to which students pay attentive interest to, participate in, and enjoy the learning activities
System maintenance & change	Educational system’s maintenance and responsiveness to change
Shared control	The extent to which students share or have the control over their learning activities’ organisation and execution
Order and organisation	The extent to which—and the way how—the learning process is organised and facilitated
*Physical learning environment*	
Naturalness	Environmental aspects affecting physical comfort and well-being
Light	The extent to which natural and artificial light contribute to health and well-being
Sound	The extent to which sound level and acoustics contribute to health and well-being
Temperature	The extent to which air temperature contributes to health and well-being
Air quality	The extent to which air contamination/freshness contributes to health and well-being
Links to nature	The extent to which views, access to nature contribute to health and well-being
Individualisation	Functional aspects supporting the learning and teaching activities of students and teachers
Fitness	The extent to which the PLE is suitable and usable, appropriately supporting the teaching and learning activities
Flexibility	The extent to which the PLE supports the (unpredictable) change of teaching and learning activities, including the effort to change
Connection	The extent to which the PLE’s configuration and connections hinders or stimulates interactions between users and their teaching and learning activities
Personalisation	The extent to which the PLE can be personalised, making the PLE distinctive and recognisably linked to the user or user group(s)
Stimulation	Configurational and aesthetic aspects stimulating learning behaviour
Expression	The degree to which the PLE's appearance evokes certain behaviour
Complexity	The degree to which the complexity of the design of the PLE evokes certain mood/feeling
Colours	The degree to which the colours used evoke a specific mood and/or behaviour

With the PPR framework, the empirical research can be analysed from an integrated psychosocial-physical perspective. In our review, we conclude that, compared with the PPR framework, recent empirical studies are often limited in their operationalisations, with the emphasis either on the PSLE or on the PLE (Baars et al., 2020). However, it can be questioned whether all subdimensions and aspects of the PPR framework are equally important for understanding how innovative PLEs are interpreted, used, and experienced by users. Our review of empirical studies has led to the following insights:

- Research confirms the relationship between *Naturalness* aspects and cognitive learning outcomes (Barrett et al., 2015). Yet, a relationship between Naturalness aspects and pedagogical practice could not be established.
- There is a growing body of evidence that *Individualisation* aspects are related to pedagogical practice, but also that this relationship is not deterministic. Innovative PLEs do not always evoke innovative practices (Beery et al., 2013), and innovative practices are also being applied in traditional PLEs (Dovey & Fisher, 2014). Many aspects seem to be involved in the construction and implementation of the pedagogical practice by the users.
- Research regarding *Stimulation* aspects is scarce and its results are ambiguous (Blackmore et al., 2011). A relationship between Stimulation aspects and the pedagogical practice has not been established.

This suggests that the three subdimensions are not equally important for gaining insight into how innovative PLEs are interpreted, used, and experienced by users. In particular, the sub-dimension *Individualisation* can be linked to pedagogical practice, but there also seem to be mediating and moderating factors.

Organised according to the PPR framework, the following paragraphs describe the relationship of this subdimension with the PSLE in more depth, as indicated by previous studies. Each paragraph starts with a description of the main relationships that have been observed, followed by the factors that appear to influence these relationships.

### PLE fitness, flexibility, connections and personalisation: what empirical research indicates

#### Fitness

Several studies examined the assumption that the PLE must fit the characteristic learning settings of innovative education as described by Thornburg (2004) and Fisher (2006, 2007). Fisher suggests applying at least three learning modalities in learning spaces: the traditional teacher-centred mode, with unilaterally oriented furniture arrangements apt for presentations; the student-centred mode, with concentric furniture arrangements apt for collaboration; and the informal mode, with casual furniture arrangements apt for informal interactions. Empirical studies confirm that such innovatively configured spaces stimulate more student-centred pedagogies (Byers et al., 2018b; Cleveland, 2016; Jorion et al., 2016). Teachers feel encouraged to change their teaching style (Byers et al., 2014) and students experience more active, collaborative, and creative learning activities compared with their peers in traditional classrooms (Byers et al., 2018a).

However, whether the PLE’s possibilities are used depends on the teachers’ “spatial competency” (Lackney, 2008) to recognise and apply those possibilities. Teachers and students do not immediately recognise the pedagogical settings that can be applied in innovative PLEs (Cleveland, 2016) because these PLEs are fundamentally different from the traditional PLEs with which they are familiar (Fisher & Newton, 2014), and because they are often unaware of the intentions of the commissioners and designers (Daniels et al., 2019). Both teachers and students have to develop new spatial competencies in a trial-and-error process (Cleveland, 2016). The ease with which users develop these new competencies varies from person to person (Mulcahy et al., 2015). Teachers who are able and willing to expand and align their pedagogy adapt quickly and benefit most from the PLE’s possibilities (Byers et al., 2018a). Cleveland's research (2016) indicates that involving teachers in discussions regarding use experiences can contribute to their understanding of the PSLE-PLE relationship. However, the construction of new pedagogical practice appears to be hampered if a team does not experience a sense of ownership of the space (Cleveland, 2016; Woodman, 2016).

#### Flexibility

As Woodman (2016) points out, the actors interpret the PLE's flexibility differently. Policy makers and architects mainly associate flexibility with physical adaptability, while teachers and students associate flexibility mainly with the PLE’s diversity and unobstructed movement. Observations of pedagogical practice show that the diversity of spaces and furniture arrangements contribute to the diversity of both teachers’ pedagogical repertoire and students’ learning activities (Byers et al., 2018b; Kariippanon et al., 2018, 2019). Teachers feel better equipped to adapt their pedagogies to individual students’ needs (Byers et al., 2014; Kariippanon et al., 2018). The open and non-hierarchical furniture arrangements promote more movement, facilitating teacher–student and student–student interactions (Kariippanon et al., 2018). Teachers experience that, without their traditional and fixed place at the front, they tend to move around more, having more interactive moments with more students. Students experience that diversity and free movement enable them to work where they feel and perform best (Mulcahy et al., 2015). In contrast to PLE’s diversity, the PLE's rearrange-ability seems to be hardly used in the pedagogical practice. Woodman (2016) observed that users rarely rearrange the furniture arrangements, although they had stated that rearrange-ability was important to them. Woodman’s research indicates that space ownership could be at play. Without sense of ownership, teachers and students don’t feel free to rearrange the furniture.

#### Connection

Research confirms that connections between spaces in a learning cluster support the flow and change of learning activities (Bradbeer et al., 2017). Teachers mention that the spatial connections between spaces in a learning cluster enable them to mix student groups and stimulate peer-learning, as well as changing group compositions in response to changing learning needs (Byers et al., 2018b). Students mention that they have better access to teachers and peers, thereby stimulating collaborative learning and socialising and enhancing the community’s cohesiveness (Mulcahy et al., 2015). However, teachers struggle to empower students to move to other places, as they experience this as a loss of control (Woodman, 2016). Dovey and Fisher (2014) suggest that maybe this is why teachers consider the visual connections important for supervising the learning processes. Students experience this supervision as inconsistent, with teacher’s expecting that they should be self-regulating. As Daniels et al. (2019) point out, teachers' perceptions of the need for supervision influence students' appreciation of visual openness. When teachers put emphasis on supervision, students dislike the openness because they feel watched, when otherwise, they value the openness for promoting social contacts.

PLE openness is also experienced as ambivalent in the teacher–teacher relationship. According to teachers, openness supports teachers’ collaboration and offers opportunities to learn from each other. However, some teachers also experience this as a threat, because others could observe their pedagogical failures (Mulcahy et al., 2015). Moreover, unlike traditional PLEs where teachers worked individually in separate rooms, teachers in innovative PLEs have to collaborate because, without coordination, the openness of the room causes acoustic disturbances between activities (Daniels et al., 2019; Kariippanon et al., 2018). Teachers have to learn how to organise the shared use of the innovative PLE (Saltmarsh et al., 2015).

#### Personalisation

Research indicates that ownership of a fixed place—where students can meet their peers and teachers for learning activities—strengthens engagement and affiliation, especially if the users are empowered to personalise their space (Cleveland, 2016; Woodman, 2016). For students, the personalisation of a space through their own action creates a strong bond; much stronger than the display of students’ projects that teachers associate with personalising the PLE. However, personalisation of a space by a specific group implicitly signals restrictions on access and use by others (Scott-Webber, 2004). This ownership of the space by a specific group can conflict with the flexible use of spaces by a learning community consisting of more groups. If other groups use these personalised spaces, this might lead to distancing and frustration with those who personalised the space (Woodman, 2016).

#### Conclusions from empirical research

The above overview gives an impression of current insights, showing that empirical studies confirm the relationships between the PSLE and the PLE’s fitness, flexibility, connection, and personalisation, but also that these relationships are ambiguous and mediated and moderated by the PLSE’s system aspects, including the sharing of control and the pedagogical practice’s order and organisation. The empirical research underlines that the PSLE-PLE relationship can only be properly understood from a relational, psychosocial-physical approach, which does not separate people (the psychosocial environment) and their physical environment, but regards them as constantly interacting with each other.

This overview shows that progress has been made in recent years, but also that additional empirical evidence is still needed. Compared with the many aspects involved, the number of studies is limited. The spread across school types, education systems and cultures is too small to draw general conclusions. Research often focuses on primary and secondary schools and is frequently part of temporary government investment programs. In addition, the results are usually descriptive, or otherwise established with different theoretical frameworks. Aspects are classified or operationalised differently. The psychosocial and physical elements with which these aspects are associated are not always identified. Without systematic ordering of the results in an overarching theoretical framework—such as the PPR framework—the studies are difficult to compare and aggregate.

## Current study

### Aim

The current study was conducted to explore the applicability of the PPR framework in empirical studies for mapping the network of interrelationships in a systematic way, as well as for gaining more insight into the dynamic construction and maintenance of the PSLE-PLE relationship by teachers and students, with identification of:

- elements of the PSLE and PLE and their meaning in this relationship (ordered by aspect—element)
- PSLE/PLE elements that mediate/moderate these relationships (ordered by aspect—element).

This study focuses on the aspects of the PLE’s sub-dimension of Individualisation (fitness, flexibility, connection, and personalisation) because these appeared in the literature as important aspects in the network of relationships between the elements of both the PSLE and PLE, together constituting the representation of the PSLE-PLE relationship, as perceived and experienced by teachers and students.

### Research questions

For this study we used the following research question and sub-questions:

Which relationships do teachers and students experience between their pedagogical practice and the physical learning environment’s fitness, flexibility, connection and personalisation?

- What meaning do they assign to the psychosocial and physical elements of their learning environment?
- Which psychosocial and physical elements of their learning environment do they identify as mediating or moderating factors?

### Methodology

#### Context of the study

The transformation of both the PSLE and PLE at the NHL-Stenden University of Applied Sciences (NHLS), Leeuwarden, The Netherlands, provided a unique opportunity to conduct this study to enhance knowledge about the relationship between innovative PSLEs and PLEs, particularly in higher education. The NHLS was created by a merger of two Universities of Applied Sciences in 2018. In this merger process, a shared pedagogical vision was formulated, based on the exchange of pedagogical experiences of both universities and steered by the literature about innovative pedagogies, including Problem Based Learning (Loyens et al., 2013), Universal Design for Learning (Hall et al., 2012) and Self Determination Theory (Deci & Ryan, 2008). This innovative pedagogical vision—Design Based Education (DBE)—is based on a social-constructivist approach combined with self-regulated, contextual and research-oriented learning within a learning community (Geitz & de Geus, 2019). DBE acknowledges that academic and social bonding appear to be predictors of study success (Jensen & Jetten, 2015; Kerby, 2015). Students—organised in groups of approximately 24—work on practice-related assignments (Gruenewald, 2003; Zandvliet, 2014) with support from a teaching team. Actual and complex issues are tackled in an iterative process in which knowledge is constructed and applied in the development of solutions to bridge the gap between the current and the intended situation. This process distinguishes different phases, analogous to the Design Thinking process: empathising, defining, ideating, prototyping, testing, evaluating and improving (Scheer et al., 2012). Together with the teaching team of 10–15 teachers, the students form learning communities related to a specific field to share and create knowledge in collaboration between students, teachers and external partners in a dialogical process (Biesta, 2013). Based on the experiences of the Design Factory at the Aalto University (Björklund et al., 2019) and of the department Built Environment at TU Delft, each learning community has its own physical place within which the learning community’s pedagogical spaces are clustered, including the workplaces of teachers and students. Because of the small-scale size of the learning community—with approximately 250–350 students using a dedicated area within the campus—and the organisation of education in groups, the policymakers expect that the students will feel socially affiliated (Deci & Ryan, 2008). In anticipation of the merger and the institute-wide introduction of DBE, various teaching teams were applying DBE already. These teams were encouraged to renovate their PLE, based on their assumptions about the requirements their new PSLE would impose on the PLE. Around 30 PLE transformations have been realised in recent years.

#### Selection of PLEs

From these innovative PLEs, six cases were selected on the basis of criteria including generalisability, representativeness, and feasibility to participate. Nine cases are located at long travel distances from the campus, which presented limitations for participation; nine cases are very highly specialised laboratories or professional working environments which limit generalisation; two cases were still in development, and two were no longer available at the time of the study. From the remaining eight, six were willing to participate. The innovative PLEs are spatially-typologically varied, from a single space to a learning cluster. The PSLEs are also varied, because the various teams incorporate the DBE approach in their own way in their PSLE, tailored to their discipline and learning community. For space plans and a short description per case, see Table 4 in the Appendix.

#### Selection of respondents

This study focused on the users of the innovative learning environment because users can provide rich qualitative data that is important for gaining a deeper understanding of the PSLE-PLE relationship (Moos, 1980). Taking into account the different representations, three focus groups were composed for each case: a focus group for students who experience the learning environment (the attained PSLE-PLE)*;* one for the teachers who put the psychosocial-spatial learning environment into practice (the implemented PSLE-PLE); and one for those teachers—mostly team staff—involved in the design of the psychosocial-spatial learning environment (the intended PSLE-PLE). The contact persons for these cases were asked to recruit respondents for these focus groups, with the request to recruit respondents with sufficient experience with the new PSLE-PLE. In two cases, the PLE is designed by the team together with the students every semester. In those cases, no separate team staff group was formed (see Table 2).

**Table 2:** Number of interviewees for each case

Case	Team staff	Teacher	Student
A	–	3	2
B	–	2	3
C	2	4	3
D	1	2	3
E	1	2	2
F	2	3	3

#### Interviews

Interviews were conducted as focus group interviews, whereby the experiences of an interviewee can be supplemented or contradicted by the others. The interviews were held in an online environment in May and June 2020, when the PLE was not accessible because of Covid-19. An interview guideline was written with an introduction, explanation of the purpose of the interview and five pre-formulated question (one question for each aspect of the sub-dimension of Individualisation) and, finally, an open question inviting the interviewees to provide information that had not yet been discussed. During the interview, the script was largely used as a checklist because interviewees already had mentioned the aspects and perceived relations of their own accord. In order to get the best possible insight into their construction of the network of relationships, the interviewer did not interrupt their train of thoughts. The interviews were recorded with the consent of the participants. After anonymous transcription, recordings were deleted.

#### Analysis

To analyse the interviews, a provisional codebook was constructed based on the PPR framework’s sub-dimensions and aspects of the PSLE and the PLE (cf. Miles & Huberman, 1994). An audit procedure was followed to test the comprehensibility, consistency and reliability of the coding (Akkerman et al., 2008). Two researchers, a lecturer-researcher from the NHLS Department of Built Environment and the first author of this article, independently coded two transcribed interviews. More than 80% of the aspects and relationships were coded in the same way. When codes turned out to be unclear, the name and description were adjusted to obtain the same understanding. Where necessary, a new code was added to the codebook, or existing codes were combined, broken down into sub-aspects, or removed. After coding two other interviews with the improved codebook, it was discussed with the research team. After that, hardly any adjustments were deemed necessary. Subsequently, the principal researcher coded the remaining interviews, and revised—with the final codebook—the previously-coded interviews (see Table 5 in the Appendix). To interpret the relations between PLE and PSLE, the relations between attributes were put into diagrams that summarise the main relationships as experienced by the interviewees. For each interview, an overview was made of these relationships and corresponding typical quotes, categorised for each aspect of the PLE’s subdimension Individualisation. A cross-case analysis (Miles & Huberman, 1994) established the relationships which were most frequently identified as being important.

### PLE fitness, flexibility, connections and personalisation: what teachers and students tell us

The following section describes the results, organised in paragraphs for each aspect. Each paragraph starts with a description of the main relationships that are experienced by the interviewees, followed by the factors that, according to them, influence these relationships.

### Fitness

#### Capacity and ergonomics: as in education, the space must be tailored to students and their activities

The interviewees associate PLE *fitness* with the *availability* and *capacity* of physical facilities, experienced as preconditional for conducting the learning activities (*teaching repertoire, learning diversity*). According to the interviewees, the *capacity* of the floor space determines how many students can be accommodated in a particular pedagogical setting, with presentation settings requiring the least space and collaborative settings needing the most space:

I’ve had someone presenting here once and it was completely packed with 120 people, crammed like sardines, but it fitted. When applying a collaborative setting with several groups, then with 25–30 people, we reach the max. of what fits.

Regarding collaborative settings, both teachers and students mentioned that knowledge sharing and knowledge construction is strongly enhanced by the *availability* of writable surfaces and displays.

Regarding their personal workplace, students mentioned the table surface’s *capacity* for the disposition of materials and equipment, and the *capacity* of power supplies for their devices. Students also note that poor chair *ergonomics* causes physical discomfort, hindering their *attentiveness and focus.* The quality of the chair is also perceived by them as a sign of respect and *inclusiveness.* Teachers agree with this, as one said:

Everyone sits on the same chair, comfortable and adjustable to one’s characteristics. Not only our educational but also our physical environment has to be designed according to the principle that every student is unique.

#### Congruency: you cannot not participate in physical innovative settings

The interviewees also associate PLE *fitness* with *visibility, intelligibility,* and the PLE’s *ergonomics*, together with the positioning of participants, constituting the PLE’s *congruency* with the pedagogical setting. They experience that these sub-aspects influence students’ and teachers’ behaviour (*teacher’s approachability*, *receptiveness/supportiveness; students’ attentiveness, focus, participation, initiative*). The interviewees note that physical encounters are crucial to establishing and maintaining relationships within the learning community. According to them, this has to do with the multi-layered communication in a PLE, with fluently changing communication settings involving formal and informal communication, intended and unintentional:

You cannot ‘not communicate’ in a physical environment, such as you can in virtual meeting: turn off the webcam and mic. Seeing is essential, but actually feeling is much more important.

Interviewees associate *visibility* with participants’ view direction, influencing students’ involvement. Students and teachers mention that, in traditional furniture arrangements, visual interactions between participants are limited, stimulating passive audience behaviour:

In open learning spaces, it is much easier to cooperate than in a traditional classroom with a bus arrangement. Then you are actually working individually and listening to one person.

In contrast, students and teachers experience that concentric arrangements, without any physical separation between the participants, lead to more interaction. As a teacher said:

For social interaction, we position students in a circular arrangement without tables in front of them, forcing them to be open to each other. Sitting behind a table makes them immediately more reserved.

The interviewees indicate that the relative physical positioning—regarding height difference, distance and the like—also influences the psychosocial positions, which can be reinforced by the furnishing, such as tables’ form and seating arrangement that indicate positions and roles of participants:

I take a central position at a rectangular table’s short side when I have to lead the discussion. With square or round tables there is always someone sitting next to you, in an equal position.

In addition, the interviewees perceive that the type of seating influences the sitting posture and thus behaviour. With informal seating, people sit back, associated with a relaxed posture that befits informal discussions and idea-forming. With formal seating, people sit upright, associated with an alert attitude, as befits formal discussions and presentations for which attention and active participation are expected. Regarding the furniture arrangement, the teachers mention that the furniture arrangement’s ‘*congruency’* with the intended behaviour is more important than in traditional pedagogies. They perceive that, unlike traditional pedagogy, innovative pedagogy requires psychosocial interaction as one of the most-important elements and that physical positioning has impact on those psychosocial interactions:

If you have a lecture, the type of furniture arrangement is actually not that relevant. But if you want to observe how the students actually collaborate, or with what attitude they tackle an assignment, then you definitely need an innovative arrangement.

#### Spatial competency: thinking about space and education has the nature of a conversation

Several interviewees noted that not every teacher is equally spatially competent (*teacher’s pedagogical repertoire’s diversity*) to assess *furniture arrangement’s congruency* with the pedagogical setting:

I think recognising the correct furniture composition requires training. Since I switched to innovative pedagogy, I apply ‘the art of hosting’. I consciously choose the arrangement that optimally supports my setting. However, some colleagues have less affinity with this.

A team leader notes that, with the realisation of the innovative PSLE-PLE, the process does not stop because innovation involves a continuous process of exchanging thoughts and experience:

That is always with innovations, a few people get to work enthusiastically and the rest a little less. I noticed that thinking about space and education is more than conveying a certain concept; it has more the nature of a conversation.

Teachers also underline the importance of team dialogue for the pedagogical practice (*team’s reflectiveness*), but also for designing an appropriate PLE. They note that the implemented *pedagogical repertoire* is determined by the teaching team’s *learning goal orientation* and that therefore that a PLE that fits well with one team does not necessarily have to fit well with other teams with different learning orientations. They also note that discussing the PLE's design deepened their discussions on pedagogy:

This is the most valuable thing that the design of the physical learning environment has brought us: thinking about and discussing how we design and organise our education: from the basics, what you stand for, and which learning environment suits that best, educationally and physically. If you advocate a different pedagogy, then you may need completely different physical configurations.

In the cases when the users have been strongly involved, a good fit is experienced between the PLE and PSLE. When the users have been less involved, or not involved at all, teachers are significantly less positive about PLE’s fitness:

It has been thought for us—with the best of intentions—by others, not with us. They have come up with all sorts of things and forgot to ask us. As a result, things have been built that don't work well at all. Because things can’t be changed anymore, we adapt to the space and not the other way around.

There are contrary experiences in the cases for which users are empowered to (re-) design and adjust the space. Here, knowledge is constantly being developed about what works and what doesn't, as a member of team F said:

We are always trying something new to support the educational activities. We also often ask students to come up with solutions. Sometimes it fails, sometimes you solve something. We learn from that. I am proud of what we have achieved here. Only you should never stop developing. There is still much to gain…

### Flexibility

#### Diversity: you can work dynamically, each place has a different function for me

The interviewees associate PLE *flexibility* with the *diversity* and *rearrange-ability* of PLE elements—ranging from the scale of a learning cluster to the scale of pieces of furniture in a space—indicating that this supports the diversity of pedagogical settings (*teaching repertoire’s diversity, learning diversity*) as well as the ability to switch quickly between pedagogical settings (*pedagogical agility*). According to the interviewees, PLE diversity can be realised by combining a *diversity* of spaces within a learning cluster, or through a *diversity* of furniture and furniture arrangements within one space. As a student from case D said:

The classrooms and the study landscape have a separate function for me. When I’m in the classroom, I’m there because then I have lessons; teacher-guided activities. When I’m in the study landscape, furnished with individual desks, I’m there to work on my assignments. We use the informal seating for discussing or just chatting.

Furniture arrangement *diversity* within one space enables switching directly between different settings as a teacher from case C said:

In the default position, you can work dynamically, using the furniture arrangements’ diversity. You can respond quickly to students’ learning needs. Above a certain number of students, you have to adjust, limiting and losing furniture arrangements’ diversity. We prefer to avoid that, because then you may lose the pedagogically dynamic quality of the space. In those cases, we propose to split into two sessions. Nevertheless, it is convenient that I can rearrange the furniture during the day, to fit the changing learning activities. I regularly do that, but I always return it to the default position because that’s the best.

#### Rearrange ability: it is a lot of hassle

Although the users say that they consider the *rearrange-ability* of the furniture arrangement important, they do not rearrange quickly. A number of factors are mentioned, including the *floor area’s fitness*, *furniture’s moveability/* *change convenience* and the *team’s spatial competency*.

Not all learning settings can be realised in the same space for the same number of students. In many interviews, the interviewees mentioned that, after a plenary start in a presentation setting, students split up into groups for elaboration in a discussion setting or a collaborative setting. Then, groups often leave because group settings require more space.

When alternative spaces are not available, the PLE’s *change convenience* determines whether its rearrange-ability is actually used. The interviewees link the PLE’s *change convenience* to the *movability* and *stackability* of furniture. Because of their weight, large furniture and walls are experienced as an obstacle to rearrangement:

You can use those wall elements to create spatial units for group work. We don't apply that much because it is a lot of hassle to move those things. They are quite heavy. If moving was really easy, we would use it more often.

Change convenience is even more decisive in cases for which teachers need to return the furniture to its original configuration after a short period of use.

Sometimes we try to change the furniture arrangement, but you often have to put everything back because another group is using that space after you. Then we don't do that because it will be at the expense of the time you want to spend on your session

For the sake of efficiency, teachers therefore do not rearrange the furniture arrangement, but rather adjust their pedagogical setting.

#### Rearrange ability: good conversations and house-rules make the difference

Where teams use a flexible PLE together, teachers underline the importance of mutual conversation and coordination, including *rule clarity* regarding rearranging the furniture (*furniture arrangement rearrange-ability*).

Teachers note that, through the joint use of spaces with a flexible layout, they become more aware of the differences within the team regarding their pedagogical preferences and competencies, including the ability to adapt to the new PLE:

From the traditional learning environment in which everything was structured and predictable, you enter a flexible environment in which you are inquisitive. New environments require an investigative and self-regulating ability—from the student, but also from the teacher and the team.

Teachers indicate that they learn in the pedagogical practice of how to use the PLE’s flexibility effectively, as a teacher and as a team, and that mutual discussions help them to strengthen their spatial competencies:

Because of the flexibility of the furniture arrangement, you get informative discussions with your colleagues about what works and what doesn’t.

In case C, the team developed rules regarding the default position in which the furniture must be replaced after use. Here, the interviewees emphasise that everyone then must adhere to the agreements, requiring a culture of *rule clarity* and *conformity*:

We address students and teachers about the use of the space. There are house rules. We point these out if they are not being followed.

However, if this culture is lacking, the PLE’s flexibility can frustrate users because they often have to rearrange the furniture from an unpredictable arrangement:

Everyone organises the furniture arrangement differently. That gives you a lot of freedom but also frustration, because when you get there, it’s never the way you want it to be.

In the cases A and B, students have a say over the PLE. Here, students emphasise the importance of mutual coordination and discussions also. They experience that without coordination a competitive atmosphere arises, If coordination does function well, they experience that this strengthens the group cohesiveness :

We made agreements among ourselves. If the agreements did not work, we adjusted them in consultation. If someone had a problem, we helped each other. At another course, agreements we made were nailed down for the entire period. There, each group claimed their own space; everyone was focused only on his own group.

### Connection

#### Proximity: meeting each other helps you to get started, to share things, to learn from each other

The interviewees associate PLE connection with the *proximity* of people in the different work/learning spaces, expressed in *acoustical, visual and physical connections*, indicating that this affects the interactions between users and thus their *relationships*.

The interviewees emphasise the importance of having a fixed area—in which students’ and teachers’ workplaces are interconnected—for fostering mutual *support*, *cooperation* and social bonding (*cohesiveness*). In this area, community members can meet, whether planned or unplanned, formally or informally. If students have questions, they can find their peers and teachers there. The interviewees underline the importance of the teacher's workplace *proximity* to the students’ workplaces in their learning landscape or ‘learning common’. In all the cases studied, teachers work in the learning common or in a space directly adjacent to it. Students also mention the benefits of getting peer support in their learning common:

The learning common is the home of the study program. If you are bothered by something, you can quickly share with others and discuss it.

Students experience that the openness of the learning common supports peer learning *(student cooperation)*:

In an open space it is very easy to talk to each other. When I discuss things, then I analyse things, adding knowledge, generating new ideas. The learning process goes much faster, because you learn from each other.

Students and teachers mention that the informal meeting place—next to the learning common where everybody walks by—promotes unplanned contact moments. According to them, these informal moments, discussing personal issues and learning issues, promote feelings of *cohesiveness* and stimulate the learning process*.* As a teacher said:

It is important to have our community spaces located together: the corridor, the hangout, the study landscape and the teaching space. For me, the best conversations and the best education take place outside the official lessons and learning spaces.

The students mentioned the importance of an informal space for social bonding also:

We were not only seriously busy there, but also having a cup of coffee, playing games. It's those little moments that make you bond with each other, stimulate you to get started, stimulate you to agree to do things together.

#### Acoustical connections: openness requires respect for disturbance sensitivity of people and activities

Although interviewees mention that connections between learning places also can be disruptive for activities requiring focus, students mention that the openness of the space is much less of a problem if the users take each other into account. As this student said:

There are no specific separations and yet the space is used enormously varied. I think it works because people do respect each other.

In addition, students also note that the level of distraction varies from person to person. Some seek out the bustle of their peers to work on their studies, while others just need silence. When working individually, students mostly use earplugs if they experience insufficient acoustical separation. Some students indicate that visual separations help them to avoid being visually distracted (e.g. sitting with their back to the others or by shielding their work place with screens).

Collaborative and discussion settings are mentioned as being most sensitive to disturbance. The interviewees indicate that this is caused by the reduced intelligibility of group members but also by distraction because of conversations conducted in the vicinity. Because of this disturbance sensitivity, groups often leave the space to find an acoustically-separated space within or outside their learning cluster for these collaborative activities.

#### Disjunction: making connections must be in the minds of people

The interviewed teachers do not experience any problems with students working outside their supervision. According to them, students have to be self-regulating and don’t need teachers’ control. Students are free to choose the place that they consider most suitable for their activities. Connections between teachers’ own workplace and those of their students are not considered very important. Moreover, educational benefits of working outside the own learning cluster (*student workplace’s disjunction*) were mentioned, such as stimulating *environmental interaction* with other study programs. As this teacher said:

We encourage students to use the spaces and facilities of the other study programs. If they get stuck, they can help each other from their own expertise.

Pedagogical emphasis on interaction appears to overcome lacking spatial connections. Even in case B, for which spaces are strongly separated, there are many interactions between the different users in the building, as one teacher said:

Making connections must be in the minds of people, then spatial separations don’t matter anymore.

According to team leaders, the position of the learning cluster can also promote or hinder environmental interaction. Team C experiences that the *disjunction* of their space from the building’s main routes hampers interaction. They say that spatial connections with a route can stimulate encounters and interaction with people, through chance encounters or by generating interest through displayed activities and projects. Whereas team C, which is focused on interaction with other study programs, prefers a location on the main route within the building, team B, which is focused on interactions with companies, prefers a location outside the campus. Their students experience that the location in the multicompany-building in the inner city promotes interaction with external stakeholders of the projects on which they are working:

On the university campus you are all in one secluded place, disconnected from society. What I really like here is that, if you want to do research, you can easily walk into the city and find people.

According to teams B and D, working off-campus is especially beneficial for senior students in their transition from education to professional practice. For younger students, having their own place at the campus is more important for building a social bond with peers and teachers who can support them in their studies.

### Personalisation

#### Customisability: by furnishing it yourself, the space becomes your own environment

The interviewees mainly associate *personalisation* with the *customisability* of a certain space or learning cluster, indicating that this affects students’ feelings of *ownership* and *affiliation*, which in turn stimulates *students’ motivation*. Teachers emphasise that, for fostering the sense of a community among learners, the learning common should be experienced as a home, and that domestic elements contribute to a homely atmosphere. Students confirm that domestic furnishings evoke their sense of community:

It feels like a safe place where I belong. I think the kitchen is important for that, but also those other domestic elements that make you feel at home and behave as you would do at home. Because everyone behaves that way, they feel like my family to me.

Teachers have different opinions regarding the PLE’s *distinctiveness* and whether this should be determined by the staff, the team or by the students. Team E has furnished their learning space distinctively and profession-specifically, because they experience that this evokes profession-specific behaviour:

For practical simulations, we prepare case studies and arrange the space in such a way that it closely resembles practice with everything that goes with it. Then that space supports professional behaviour. This style is characteristic for our profession, making our PLE distinctive.

However, such specific furnishing by the team can also hinder the students’ sense of *ownership*, as a student from case E said:

The design is a bit over the top. It doesn’t really feel my ‘own’, it feels even emptier and more clinical than it used to be when it was still a normal classroom, decorated by ourselves.

Therefore, teams of the cases A, B, and F encourage their students to furnish the PLE themselves, because they have experienced that this contributes to a *group’s cohesion* and *student engagement*. Students experience this also:

We arranged and maintained the space ourselves, in mutual consultation. By doing so, the entire space becomes your own environment. We have placed domestic elements such as a sofa, a rug, table lamps, plants. Doesn’t feel like being at school. Sitting on the couch with your friends, building a bond you would never have had in a classroom. That stimulates me to pursue my studies.

#### Customisability: it’s not so much about furnishing, it’s more about trust and having a say

According to the teams, developing feelings of *ownership* is only possible if the users have *shared control* over their PLE by not only having a say about their furnishing, but also about their PLE’s use. A student said:

It felt like our own space when we furnished it ourselves with benches and a cupboard. But then other groups were also scheduled there, and I was not welcome at that moment. After that, it no longer felt proper and safe, not being our own, anymore.

For students, personalisation has more to do with being trusted and sharing control over the PLE, than with distinctive features or style:

For me, personalisation is not so much about exhibiting my own projects, but more about the respect and trust you get and the free use of the spaces and facilities.

However, students’ empowerment and the PSLE-PLE’s scale seem to be related. A teacher of case D noted that self-management did not appear feasible anymore after the enlargement of their learning cluster, with more groups using that PLE.

### Elements, aspects, and their attached meaning in relationships

According to the interviewees, by discussing their innovative PLE with the four PLE aspects as a reference, all relevant relationship were addressed, demonstrating the benefits of applying the PPR framework. Many attributes and relations were mentioned in the interviews. With the help of the PPR framework, relations between attributes could be systematically mapped and analysed, providing insights into users’ construction of the PSLE-PLE relationship (see Table 3).

**Table 3:** PLE attributes connected to experienced relationships

Element	PLE sub-aspect	PLE aspect	PSLE aspect	Experienced relation ^a^ (incl. Mediating, moderating aspect)	
*General*					
Peers’, teacher’s	Visibility (to each other)	Fitness	Student cooperation, student involvement	Multi-layered communication, interaction	
Teachers’	Visibility (to students)	Fitness	Teacher support	Teacher’s approachability	
Students’	Visibility (to teachers)	Fitness	Shared control, teacher support	Teacher’s supervision, noticing need for learning support (teacher’s positiveness, supportiveness/receptiveness)	
Peers’, teacher’s	Intelligibility	Fitness	Student cooperation, student involvement	Multi-layered communication, interaction	
*Learning cluster*					
Learning cluster	Proximity to city	Connection	Environmental interaction	Interaction with external stakeholders, companies, organisations	
Learning cluster—logistic route	Junction	Connection	Environmental interaction, relevance integration	Interaction with students, and teaching teams of other learning communities, study programs, disciplines	
Learning cluster’s spaces	Availability/capacity	Fitness	Order and organisation	Size and number of groups and (simultaneously conducted) learning activities	
Learning cluster’s spaces	Diversity	Flexibility	Order and organisation	Variety of teaching team’s pedagogical repertoire, and student learning diversity (spatial competency)	
learning cluster’s spaces	Physical proximity	Connection	Order and organisation	Teaching team’s pedagogical agility (only in case of quickly and unpredictably changing between activities)	
Group’s meeting room	Availability, capacity	Connection	Student cooperation	Enabling collaborative activities without disturbing others / being disturbed by others	
Learning common—teacher workplace—informal meeting	Proximity	Connection	Teacher support, student involvement	Stimulating and enforcing mutual personal relationship between students, and between students and teaching team members (community’s ownership)	
Spaces separation (wall, room dividers)	Rearrange ability, adaptability	Connection	Order and organisation	Increasing the possibilities to rearrange groups and/or activities, and moving of students and teachers (change convenience, empowerment)	
*Space*					
Floor area	Capacity	Fitness	Order and organisation	Group’s size, size and number of sub-groups and (simultaneously conducted) learning activities	
Space’s design	Distinctiveness	Personalisation	Group cohesiveness	Feelings of belonging to the place and community	
Space’s furniture arrangement and finishing	Profession-similarity	Expression	Student cooperation, student involvement	Specific cultural/professional behaviour	
Furniture arrangement	Congruency	Fitness	Student cooperation, student involvement	Behavioural patterns of the intended pedagogical setting (spatial competency)	
Furniture arrangement	Diversity	Flexibility	Order and organisation	Variety of teaching team’s pedagogical repertoire, and student learning diversity (spatial competency)	
Furniture arrangement	Rearrange-ability	Flexibility	Order and organisation	Variety of team’s pedagogical repertoire, student learning diversity, pedagogical agility, (spatial competency, space’s floor area, empowerment to rearrange, rule clarity)	
Student’s workplace	Separateness—peers’ workplaces	Connection	Student cooperation, student involvement	Interaction with peers, either disrupting or enhancing students’ learning activity (student respectfulness, rule clarity & conformity)	
Student’s workplace	Separateness—teacher’s workplace	Connection	Teacher support	Interaction with teacher (e.g. For answering learning needs)	
*Furniture, finishings & equipment*					
Furniture	Ergonomics	Fitness	Student involvement	Students’ focus, student’s feeling of being respected, included	
Furniture, power supply, equipment	Capacity, availability	Fitness	Order and organisation	Teacher’s pedagogical repertoire, student learning diversity	
Display (e.g. Writable surfaces, boards)	Availability	Fitness	Student cooperation	Exchange of ideas	
Furniture, equipment	Diversity	Flexibility	Order and organisation	Variety of teaching team’s pedagogical repertoire, and student learning diversity	
Furniture, equipment	Move-ability Stack-ability	Flexibility	Order and organisation	Pedagogical agility, pedagogical repertoire (spatial competency, empowerment to rearrange)	
Furniture, equipment	Homeliness	Personalisation	Group cohesiveness	Feelings of belonging to space and community	
Furniture	Customisability	Personalisation	Order and organisation	Feelings of ownership (empowerment)	
Furniture, equipment	Controllability lockability	Personalisation	Student involvement, shared control	Students’ feelings of ownership	
Product display	Promotionality	Expression	Environmental interaction, relevance integration	Raising curiosity of passers-by, stimulating interaction and involvement	

^a^In this overview, relationships are abstractly described. For a deeper understanding, see the corresponding paragraph

## Discussion

This study was conducted to show the value of the relational psychological approach in examining innovative learning environments and the usefulness of systematically mapping the PLE-PSLE relationship using the PPR framework. By examining this relationship through the lens of the PLE aspects of fitness, flexibility, connection and personalisation, new insights were gained into the relationship as experienced by the students, teachers and team staff. Overall, networks of relationships as identified in the theoretical background were confirmed, clarified, and nuanced by the systematic analysis based on the aspects as included in the PPR framework, as well as paying attention to the three different representations.

Although the current study was focused on user experiences in higher education, many observations are consistent with previous empirical research which mainly focused on other educational levels. The results confirm the importance of the ongoing discourse about the PSLE-PLE relationship, between the actors involved in the various levels of the school system, and also between the levels: between teachers and students, between teachers in teaching teams, between teams and the central staff, and also between these users and external advisors involved in the PSLE-PLE transition. The results also show that, during the transitions—and afterwards—changing aspects entail rebalancing the system aspects of power and control. Compared with the studies discussed in the theoretical background, this study revealed some differences which might be explained by the age of the students and the larger organisation of a university. However, the results also shed new light on the Individualisation aspects—fitness, flexibility, connection, and personalisation—related to the PSLE, as well as the factors that mediate and moderate these relationships.

The following paragraphs describe the relationships of the PSLE with the PLE aspects of fitness, flexibility, connection, and personalisation in more depth, comparing the results of this study with previous studies. Each paragraph starts with the main relationships, followed by the factors that appear to influence these relationships.

### Fitness

#### Multi-layered interactions require appropriate physical environments

The necessity of the availability and capacity of PLE elements mentioned by the interviewees corresponds to the spatial requirements as formulated by Fisher (2005). The interviewees’ perceptions also support the assumption that furniture arrangements influence users’ behaviour and thus the pedagogical setting (Scott-Webber, 2004). The interviewees noted that the experience of fitness of arrangements strongly depends on the teams’ implemented pedagogy. They mentioned the innovative pedagogies being most vulnerable. This observation takes on additional meaning because it is based on the experiences of the teachers and students during the period when the Covid measures limited the interactions to virtual contacts. Both students and teachers experience that a virtual environment reasonably supports singular interactions, such as in the presentation setting and the one-on-one setting. But, according to them, this does not apply to the interactive settings that are characteristic of innovative pedagogy. Because of the lack of a physical learning environment, they felt severely limited in their innovative pedagogy. This underlines the importance of the PLE for—and its alignment with—innovative educational practices.

#### Experiencing fitness requires empowerment of users in design and implementation

Alignment about the intended use of the PLE—as intended by the user and as foreseen by the designer—appears essential for the experienced PLE's fitness for users' pedagogical practice (Daniels et al., 2019; Lippman, 2010). The current study suggests that the degree of involvement in the design correlates with the experienced fitness. Similar to Woodman's (2016) observations on empowering users in the design, the current study shows that those who co-designed their PLE feel spatially engaged and eager to share their knowledge regarding the pedagogical use of the new PLE with others. Thus, the space might not be a change agent as such, but the users involved in changing the space are the true agents of innovation, which seems to be overlooked by policymakers.

#### Maintaining fitness requires continuous discourse

All interviewees were eager to acquire and share knowledge and their experiences, positive and negative alike. Because students determine their own learning space for most of the time, they discuss the fitness of places for their common learning activities almost daily. Based on their experiences, they take into account the nature of the activities and the personal differences. The interviewed students showed ‘spatial competency’, which is consistent with Woodman’s (2016) observation that students who are allowed to self-regulate their PLE show more spatial competency and engagement. Teachers also mention that discussions with their colleagues contribute significantly to their understanding of the PSLE-PLE relationship. Additionally, they mentioned the added value of training and expert advice on demand. This underlines the importance of preparing teachers to use innovative PLEs (Byers et al., 2018b), but also the importance of stimulating teachers’ and teams’ continuous reflectiveness (Bradbeer et al., 2019).

### Flexibility

#### Rearrangement requires effort and time

The perceptions of the interviewees regarding PLE flexibility are in line with earlier research indicating that teachers and students associate PLE flexibility primarily with diversity—either in the same space, or by moving to another space (Woodman, 2016). They perceive that the PLE’s diversity contributes to teachers’ pedagogical diversity and students’ learning diversity (Byers, et al., 2018a, 2018b). PLE’s rearrange-ability is hardly used. This study indicates that students’ preference could be explained by their estimation of the effort and time needed to change, weighted against the time for which the changed PLE will be used and its estimated impact on the learning. That might also explain why users sometimes prefer to adapt their learning or teaching to the PLE, rather than using the PLE’s flexibility for adapting the PLE to their PSLE.

#### Flexible PLEs require flexible PSLEs

Empowering users to adapt their PLE to their educational needs might solve this hindrance. In line with observations in earlier research, the interviewees mentioned that, if users are empowered to adapt their PLE, good coordination is needed (Kariippanon et al., 2018; Saltmarsh et al., 2015). The interviewees’ experiences indicate that, in those cases for which the PLE’s maintenance and change are organised clearly, the PLE’s flexibility functions the best. The current study shows different ways of organisation: by mutual agreements about the return to an optimal standard furniture arrangement, by appointing one team member who manages the furniture arrangement and advises users, or by procedural agreements on mutual coordination. In the cases of perceived inadequate coordination or non-compliance with the rules, PLE flexibility frustrates users and therefore users even avoid that PLE, indicating that rule clarity and conformity regarding the use and re-arranging of the PLE is of the utmost importance.

### Connection

#### Feeling connected requires interconnected core spaces

The interviewees emphasised the interconnectedness of the workplaces for teachers and those for students, and a meeting place for informal activities, which together constitute the ‘home base’ for reinforcing group cohesion (Woodman, 2016). The cases included in this study show that—depending on the PSLE’s organisation—this home base can have the scale of one space used by one student group, up to the scale of a spatial cluster used by a number of groups forming a learning community.

#### Open spaces require supportive minds

The interviewees mentioned that the PLE’s openness stimulates teacher support and student cooperation, as observed by Byers et al. (2018b). However, openness can become an Achilles’ heel if the PSLE’s organisation does not take into account the limitations of visual and audial connections. Interviewees mentioned coordination of activities and a culture of mutual respect and supportiveness as being crucial for open PLEs, which confirms observations of research on open PLEs at other education levels (e.g. Kariippanon et al., 2018).

#### Interaction requires, foremost, open minds; connections help, but matter less

In most cases, the spaces of the clusters are physically separated from each other with non-convertible walls. Contrary to the assumptions of Dovey and Fisher (2014) regarding the openness or convertibility of separations between learning spaces, the interviewed teachers don’t experience the walls as limitations to the flow of people and activities to other spaces within the cluster. These findings add nuance to the assumptions regarding the necessity of spatial connections between all learning spaces within a cluster, as incorporated in the innovative spatial models of the conceptual literature.

### Personalisation

#### Personalisation requires ownership

The student experiences as recorded in this current study are in line with Woodman's (2016) conclusion that students' ability to organise and control the space themselves contributes to their ownership of the learning environment, social cohesion and thus their study motivation. The current study indicates that this also applies to teachers. For both students and teachers, the empowerment to personalise and manage their own space contributes to their engagement, motivation and satisfaction.

#### Empowerment is connected to scale

The current study also shows that violation of this empowerment is perceived by both students and teachers as devastating for their feelings of ownership. Personalisation and ownership seem to be inextricably linked. However, self-management is bound to scale. This study indicates that the scale of the space—and thus the size of the group that owns that space—entails different scales of personalisation. With a small space and a small group, the space can be used, managed, and personalised by one group, which creates a strong bond. However, if the cluster and number of users is larger, and the same spaces are used by multiple groups, frictions arise if one group psychosocially claims a space through personalisation. Personalisation appears to be connected to the ownership of the total community of teachers and students who use that particular PLE. The larger the community is, the larger is the PLE, and the more impersonal the personalised elements tend to be.

### PSLE-PLE in transition: rebalancing the power and control in relationships

The above descriptions of the four PLE aspects and their mediating and moderating factors clearly reflect the complexity of the experienced relationships. Users do not experience segregated relationships, but a network of relationships between aspects for which changing one aspect has consequences for the whole network, like a spiderweb in transition. The interviewees regularly referred to their pedagogical goals and principles as a guiding benchmark for their choices and opinions; the core of their network. The system aspects of control, order and organisation are often referred to as facilitating preconditions that are connected to all other aspects. According to the teachers and students, misalignment of these system aspects with the intended PSLE can be very disruptive to their pedagogical practice, which confirms the image of the spider's web of interrelated relationships (Van den Akker, 2013), all centred on students’ development, all connected by the system organisation (OECD, 2013), and in constant flux because of changing aspects attributable to external factors or advancing insight (Daniels et al., 2019; Mulcahy et al., 2015). In our study, the open-endedness of students’ development does not seem to be up for discussion; on the contrary, it comprises the orientation point for teachers and students alike. The often-experienced friction is related to the misalignment of the system organisation, especially the distribution of control in different relationships. These experiences match the research of Daniels et al. (2019) who stated that different distributions of control can lead to opposite experiences of the same PLE aspect. For example, the current study shows opposing experiences and perceptions regarding:

- the PLE’s fitness—appearing to depend on users’ involvement in the PLE’s maintenance and change
- the PLE’s flexibility—appearing to depend on mutually discussed and maintained rules
- the PLE’s connections—appearing to depend on teachers’ empowerment of students
- and the PLE’s personalisation—appearing to depend on the empowerment of teams and students.

As the interviewees indicate, the psychosocial environment is not static but develops in interaction with the innovative PLE to solve the experienced frictions. In this process, new balances are sought between control and empowerment in the various relationships—teacher–student, teacher–teacher, and teaching team–management—including the alignment of these new balances into the system’s organisation at different levels.

#### Relationship: teacher–student

Research regularly shows that teachers have difficulties finding a new balance between stimulating students’ self-regulation and teachers’ supervision (Woodman, 2016). This would explain teachers' preference for an area of clustered spaces, which provides an overview of all student activities (Dovey & Fisher, 2014). However, as our study reveals, teaching teams who put students’ self-regulation central do not have the need for supervision. A reversal of findability and visibility manifests itself: teachers must be findable and approachable for students who have learning questions. The conclusion might be drawn that students’ self-regulation or pedagogical empowerment consequently leads to spatial empowerment of students, which sheds a new light on the innovative spatial models of the conceptual literature.

#### Relationship: teacher–teacher

Innovative learning environments require a different organisation of education in which teachers no longer provide education as individuals in a closed space, but as a collaborating team often in one and the same space. Aspects of the subdimension *Relationships* also are applicable to the psychosocial environment of the teaching team. Team leaders’ support, team members’ cooperation and cohesion appear to be essential for the team's self-regulation and self-learning capacity, which is nourished by continuous discourse featuring mutual exchange of experiences and attribution of meanings to the PSLE-PLE relationship. This indicates that team members should operate according to the same principles of social constructivism that they apply to their students.

#### Relationship: management: teaching teams

The current study underlines the importance of assigning and attuning control over the PLE between the policy level—of managers and supporting staff—and the operational level of the teams. The users regularly mentioned the frictions that arise when adjustments are required at a level of policy and management, which is beyond their sphere of influence. Where coordination and agreements within a learning community about the use of PLE by others are overruled by the central planning or facility management, users feel less connected with that PLE, which makes them feel less stimulated to use the PLE optimally (Cleveland, 2016).

This study also underlines the importance of involving users in the design of their PLE. As Daniels et al. (2019) argue, if policy makers do not involve users in the discussion about the design, architects do not become aware of the experiences and preferences of the users, and users are unfamiliar with the intended use as assumed by the designers. This can result in dysfunctional environments. Conversely, when users are closely involved in the design—or constantly redesign the environment themselves—they feel stimulated to contribute to the discourse regarding the PSLE, the PLE, and the mutual relationship between the two. Our research indicates that, by empowering users in the design process, interventions can be less invasive, resistances to processes can be reduced, and the PSLE-PLE will be used more effectively. Empowered users appear to be motivated to actively contribute to the discourse on the PSLE-PLE relationship, including conceiving, sharing, implementing, and evaluating innovations of their PSLE-PLE, not only during the design phase but also during implementation. Through continued involvement and empowerment of users, the PLE’s fitness can be ensured sustainably.

### Limitations of this study: suggestions for further research

The current study has several limitations that can be regarded as a limitation to the depth of understanding and the breadth of generalisation: the singular focus on users; the small number of cases (all within one university); the exceptional situation of Covid restrictions during the interviews; and the limitation of collecting perceptions only. These are briefly discussed in the following paragraphs with suggestions for further research.

Empowerment of the users in the process of design and management of the PSLE is determined by policy and management. Like much recent empirical research, the current study focused on innovative pedagogical practices and only the users were interviewed. This gives a limited perspective on the organisation of the system management and change. The current study did not examine the views of those enacting the processes of maintenance and change of the PSLE-PLE, including policy makers and facility managers. Given the power that these actors have regarding the distribution of control over the PSLE-PLE, it is important to explore their views in further research for a better understanding of the PSLE-PLE relationship. This applies especially to their incentives, beliefs and intentions regarding how the system organises the design and maintenance processes of the PLE, and how this contributes to the intended PSLE-PLE relationship.

As Tassone et al. (2021) mention in their research, higher education is rapidly changing, with many educational institutes involved in course innovation processes similar to those of the NHLS. Expanding our research into the consequences of these course innovations for the PSLE-PLE relationship at multiple institutes would be highly desirable to limit the influence of local effects.

The current study was entirely based on the experiences of teachers and students with their transition to an innovative PSLE-PLE, while acknowledging their awareness of the environmental aspects when changes occur. Due to Covid-19 restrictions, they went through an additional transformation because the PLE was no longer available. The unique contribution of a physical context to education became very explicit for them. They might have overemphasized the importance of PLE because of limitations that they experienced. However, comparison with other studies does not provide any indication of this.

Last but not least, gathering quantitative data involving the actual use through observations could have further deepened the insights, especially because opinions expressed do not always exactly correspond to actual use (Woodman, 2016). With such a multi-method research design—collecting perceptions and observations—whether the PPR framework is helpful in the systematic collection and analysis of both quantitative and qualitative data also could be tested.

## Conclusion

The results of the current study demonstrate the possibilities of using the PPR framework in qualitative research to provide insight into the complex network of relationships as recognised in a psychosocial-physical relational approach. The study confirms, clarifies and nuances results from previous research into innovative learning environments regarding the relationships between PSLE aspects and PLE aspects of fitness, flexibility, connection, and personalisation. The PPR framework appears to be useful for systematically identifying and analysing these relationships—including the related PSLE and PLE elements—as well as the mediating and moderating relationships of PSLE aspects, especially those of PSLE system maintenance and change. The study further underlines the importance of the ongoing discourse—between actors at the same level and between actors at different levels in the school’s organisation—for the attunement of the system aspects (shared control, order and organisation) to the pedagogical and spatial changes:

- at the scale of the learning community, for a continuous cyclical process of meaning making, implementation, experiencing and reflecting on the PSLE-PLE relationship
- at the scale of the school’s organisation, because teachers and students regularly experience that they are insufficiently empowered by policy makers and managers to implement the changes properly.

In line with recent empirical research, the findings of this study confirm that changing from a rational approach to a psychosocial-physical relational approach in designing and implementing learning environments leads to different processes and outcomes. Interventions could be less invasive, resistances to processes could be reduced, and the innovative learning environment could be used more effectively. This argues in favour of paying more attention in the research field to the actors and the level involved in shaping these processes and products: the level of the policy makers and their advisors, and the discourse through which they construct the intended psychosocial-physical learning environment.

## Appendix

See Tables 4, 5.

**Table 4:** Overview cases

*A*	

Fitness	At the start of the semester, the students may furnish the empty space themselves. In addition to standard movable furniture, they are allowed to bring their own belongings on the condition that they are removed at the end of the semester
Flexibility	The furniture arrangement changes many times, rearranged by the student to fit their activities
Connection	Teachers work in the same space as the students Students use this space as their home base, but also chose to work at other places not directly connected with this space
Personalisation	Students personalise the space with their furniture, decor and projects, regularly replacing the latter
Management, ownership	The room is used by this student group only, managing the use themselves in consultation with the teachers
*B*	

Fitness	The retrofit of this space in a historical building is designed by the teaching team. At the start of the semester, students bring in extra furniture in addition to the standard movable furniture, on the condition that they remove it at the end of the semester
Flexibility	The furniture arrangement changes every now and then, rearranged by the students and teachers to fit their activities
Connection	This is one of a number of learning spaces in a multi-company building, housing student groups of the university and vocational education, the public library and creative industry companies Teachers work in the same space as the students Students use this space as their home base, but also work at other places, such as in the public library The spaces have no visual connection with each other, the central staircase forms the only connection between the spaces The building is located a few kilometres from the campus, in the inner city
Personalisation	Students personalise the space with their furniture, decor and projects, regularly replacing the latter
Management, ownership	The space is scheduled for one group most of the time, but it is also used by other groups
*C*	

Fitness	The interior design is made in close cooperation with the team leaders. The design is regularly discussed in the team and adapted in consultation. The experiences gained here are used in the design of the new learning cluster at another position
Flexibility	The default furniture arrangement offers a diversity of learning modalities. All furniture can be moved easily, enabling quick rearrangement, under the condition of rearranging to the default position at the end of the day
Connection	There is always one of the teachers present in this space Students work and learn partly on the job, and at other spaces within the building. They use this space as home bases for their learning activities. For disturbance sensitive activities, a separate space is available The space is difficult to find and access, located in a remote position
Personalisation	The team displays various products/projects in display cabinets
Management, ownership	The space is allocated to the team and to the students taking the course. The team manages the use of the space
*D*	

Fitness	The end users have had limited involvement in the design. There was some coordination via stepped representation, but this mainly concerned the design of the teachers’ workspace
Flexibility	The default furniture arrangement offers a diversity of learning modalities. Most furniture can be moved by the users, except for the fixed walls and stands in the studio or ‘atelier’ spaces
Connection	Some teachers choose to work in the “learning landscape” next to the students, while others choose to work in the separated teachers’ workspace next to the learning landscape Student groups start in the ‘atelier’ space on fixed moment. After the start up meeting, they choose their own place to work, mostly in the learning landscape Most of the learning spaces used by this academy are included in this cluster. Walls are semi-transparent, providing a view of activities inside and outside the spaces Senior students work and learn off-campus most of the time. Students use this space as home bases for their learning activities at the university
Personalisation	For the teams and students, there are limited possibilities to personalise the space. The learning cluster is maintained by the central facilities staff
Management, ownership	The learning cluster is assigned to the teams and the students of the Academy. Spaces are assigned to teams by the central planning staff
*E*	

Fitness	The end users have had some involvement in the interior design, via the possibility to submit wishes and requirements, and via stepped representation The design is sometimes discussed in the teaching team. The experiences gained will be used in the design of following projects
Flexibility	The furniture arrangement offers a diversity of learning modalities. All furniture can be moved by the users, offering an abundance of furnishing options. Every teacher rearranges the layout according to his own insight, there is no default layout With a heavy partition wall, the space can be divided in two
Connection	Teachers work in the separated teachers’ workspace next to the learning space Student groups have teacher guided activities in this “atelier” space on fixed moments. Besides these activities, they choose their own place to work, mostly in the adjacent learning landscape. Students use the informal meeting area as home base for their learning activities at the university The coffee corner, copy machine and informal meeting area connect the learning space, general learning landscape, and teachers’ workspace
Personalisation	The distinctive character of the space is determined by the interior designer in consultation with the representative of the team. For the team and students, there are hardly any possibilities to further personalise the space
Management, ownership	The central planning staff basically allocates the learning space to the team and the students, but the space is also allocated to other teams
*F*	

Fitness	The interior design is developed in close cooperation with the team leaders, teachers and students, and is continuously in development, in a learning-by-doing process
Flexibility	The furniture arrangement offers a diversity of learning modalities. All furniture can be moved by the users, offering an abundance of furnishing options. Each teacher or student group can adapt the layout to the desired learning setting, assisted and advised by the specialised team member, who is also the space manager
Connection	The team works in the same space as the students Student groups have teacher guided activities here, but they are free to work here also at other times. Students use the informal meeting area as home bases for their learning activities at the university
Personalisation	In consultation with the learning space manager, almost every lay-out is possible. The space is full of own creations and special furniture
Management, ownership	The space is available to all employees and students of the university of applied sciences, and is managed by the learning space manager

**Table 5:** PPR codebook

		Psychosocial learning environment	
Subdimension	Aspect	attributes = element^a^ + (sub)aspect	Description attribute
Personal development	Open endedness	Teachers' learning goal orientation	The extent to which the teacher is focused on predefined uniform learning products or on open ended, personalised learning processes and outcomes
		Students' learning goal orientation	The extent to which the student is focused on prefixed learning products or on open ended, personalised learning process and outcomes
	Relevance integration	Content's multi-disciplinarity	The extent to which learning content relates to students’ out-of-school experiences, integrating specific disciplines in multi-disciplinary learning content / courses
		Students' affinity	The extent to which the student has affinity with the learning content, recognising its relevancy
		Students' curiosity/motivation	The extent to which the student is intrinsically motivated, is anxious to learn, showing curiosity / interest in the learning content
		Students' enjoyment	The extent to which the student shows positive mood and liveliness, not boredom, enjoys learning
	Environmental interaction	Teachers' environmental engagement	The extent to which the teacher/team involves the professional field or community into activities
		Students' environmental engagement	The extent to which the student is engaged in field- or community-based learning experiences, aiming to meet environmental needs with the learning products
		External partners’ engagement	The extent to which persons or organisations outside the study’s learning community (other studies’ experts, professionals, commissioners,) are engaged in learning
Relationship	Teacher support	Teacher's approachability	The extent to which the teacher is approachable to the student, is open to learning questions
		Teacher's inclusiveness	The extent to which the teacher helps students without discrimination, accepting all, treating them as individuals, being there for everyone
		Teacher's friendliness	The extent to which students perceive the teacher as a friend, a partner, being "close" to the students
		Teacher's supportiveness	The extent to which the teacher encourages, supports, praises, and helps students, explains what they don’t understand, and tries to solve issues that concern students
		Teacher's positiveness	The extent to which the teacher holds positive views for students
		Teacher's receptiveness	The extent to which the teacher is receptive—or eager—for student's comments, engaging in dialogues, not in monologues
		Teacher's inducement	The extent to which the teacher enjoys challenging students to get the best out of them, not being satisfied with minimal positive results
	Student cooperation	Students' cooperativeness	The extent to which the students cooperate, developing and discussing ideas for decision making and knowledge construction
		Students' responsibility	The extent to which the students coordinate, conduct and monitor their tasks and responsibilities as a group
	Group cohesiveness	Students' mutual respectfulness	The extent to which students accept and understand each other, not making fun of—and not discriminating, in any form—other students
		Teacher’s psychosocial commitment	The extent to which teacher(s) feel responsible for developing positive relationships in their learning environment
		Students' supportiveness	The extent to which students mutually support each other and look to benefit each other
		Students' friendliness	The extent to which students maintain friendly relationships without aggression, relaxing together / having informal activities in their free time
		Students' affiliation	The extent to which students feel themselves welcome and connected to the learning community, are 'close' with each other, knowing each other well and taking care of each other
		Students' competitiveness	The extent to which students compete in a constructive way (without jealousy), stimulate each other by sharing and comparing achievements
	Student involvement	Students' attentiveness	The extent to which students' interest and attention is directed toward the group, watching and listening
		Students’ focus	The extent to which the student is focused on the performance of the learning task
		Students' participation	The extent to which students participate in discussions; being active during the learning process, showing their will to participate
		Students' initiative	The extent to which students collect and share information on their own initiative, submitting subjects
System maintenance & change	Shared control	Student's autonomy/self-regulation	The extent to which students regulate their learning (PSLE and PLE) as individuals, and as a group, by applying their preferred way of learning (learning setting)
		Teacher's empowerment of students	The extent to which the teacher supports and empowers students to regulate their learning (PSLE and PLE)
		Team’s/teacher’s/students’ ownership	The extent to which students, teachers and/or teams feel/claim ownership of a specific physical area (territorialisation)
	Order and organisation	Team’s/teacher's rule clarity, transparency, consistency	The extent to which the team/teacher communicates the culture and rules, acting in accordance with -and maintaining—those rules
		Team’s/teacher’s organisational transparency	The extent to which the team/teacher offers a well-structured program, including clarity regarding the learning outcomes expected
		Student’s rule familiarity and behavioural conformity	The extent to which students know which (learning) behaviour is expected, and which procedures have to be followed/complied with
		Student's learning diversity	The extent to which students apply different ways of learning
		Teacher's pedagogical repertoire diversity	The extent to which teachers apply different ways of teaching/teaching settings
		Teacher’s spatial competency	The extent to which teachers recognise and apply the PLE’s possibilities for their pedagogy
		Teacher's/team's pedagogical agility	The extent to which teachers agilely tailor the learning setting to the (changing) student(s) learning needs and preferences
		Learning activities’ simultaneity	The extent to which different learning settings are applied simultaneously
		Learning activity's disruption-sensitivity/confidentiality	The extent to which an activity is sensitive to disruption
		Physical learning environment	
Subdimension	Aspect	attributes = elements + (sub)aspect	Description attribute
	Light		
Naturalness	Sound		
	Temperature		
	Air quality		
	Links to nature		
Individualisation	Fitness	Capacity (availability)	The extent to which the size and/or number of a PLE element (spatial cluster, space, furniture arrangement, piece of furniture, technical equipment) provides sufficient surface/power/quantity/etc. for the intended use
		Visibility	The extent to which users can see/discern/observe other users and objects
		Legibility	The degree to which users can read text and figures as presented on display surfaces
		Intelligibility	The extent to which users can hear and understand each other
		Congruency (with the intended learning setting)	The extent to which the shape and finishing of the PLE element (including space, furniture arrangement, furniture, technical equipment) supports the intended learning setting
		Vulnerability	The extent to which PLE elements’ finishing (including wall, floor, furniture, technical equipment) supports the intended use
		Accessibility	The extent to which easy, unobstructed movement (into and through space) or use (of tools and equipment) is supported
		Ergonomics	The extent to which the PLE element (seats, surfaces) supports the user’s body efficiently and comfortably during an activity
	Flexibility	Diversity/versatility	The extent to which a PLE offers a diversity of physical elements (including spaces/furniture arrangements/furniture/technical equipment) supporting a variety of use/learning settings
		Adaptability	The extent to which the same PLE element can be used in different ways
		Rearrange ability/moveability	The extent to which a PLE element (including furniture arrangement, furniture, technical equipment) can be rearranged by moving and/or remodelling (including link ability, nest ability) to support the change of learning activities
		Change convenience	The amount of effort and time required for changing the PLE
	Connection	Acoustic separateness	The extent to which spaces are acoustically connected or separated
		Visual separateness	The extent to which spaces are visually connected or separated
		Physical separateness	The extent to which spaces are physically connected or separated
		Temporality	The degree of temporality of the separation between spaces
		Proximity	The extent to which the distance between (inter)related spaces within a spatial cluster supports or hampers interaction
		(Dis)junction	The extent to which spaces’/clusters’ positioning (related to other spaces or logistical structure) supports interaction
	Personalisation	Controllability	The extent to which PLE elements (including climate system, light, shading, technical equipment) can be adjusted by users
		Lockability	The extent to which spaces can be locked by users
		Customisability	The extent to which PLE elements’ appearance (including a space’s finishing, displays, furniture) can be customised by users
		Distinctiveness	The degree to which PLE elements (including style of finishing, furniture and equipment) of an area used exclusively by a specific group of users—differs distinctively from style(s) of other areas
Stimulation	Expression	Natural-behaviour stimulation	The degree to which the physical spatial pattern (positioning of persons, sightlines) evokes a specific natural behaviour pattern
		Professional-behaviour stimulation	The degree to which PLE’s similarity with a specific professional environment evokes corresponding specific professional behaviour
		Homeliness	The degree to which the PLE has homey atmosphere and elements, evoking home/community behaviour
		Promotionally	The extent to which PLE elements arouse curiosity and stimulate the will to learn more about the promoted content
	Complexity	Harmony	The extent to which the form diversity and congruency of physical elements applied in the PLE evokes feelings of (dis)pleasure or unrest/peace
	Colours	Composition	
		Saturation	

^a^**PLE elements**

General (e.g. peers, teacher, students)

Campus

Building

Learning cluster

Space

Building elements (e.g. walls)

Furniture arrangement

Furniture, finishings & equipment (e.g. chair, table, display, power supply)
